# Supplementary material for: OpenMM 7: Rapid development of high performance algorithms for molecular dynamics
Source: PLoS Comput Biol. 2017 Jul 26;13(7):e1005659. doi: 10.1371/journal.pcbi.1005659 (PMC5549999; doi:10.1371/journal.pcbi.1005659)
Supplement: S1 Source Code — (ZIP) [file pcbi.1005659.s002.zip › OpenMM-7.0.1-Source/docs/api-python/generated/simtk.openmm.openmm.TabulatedFunction.html]

TabulatedFunction — OpenMM 7.0.1.dev-5e86c4f documentation


# TabulatedFunction¶

*class* `simtk.openmm.openmm.``TabulatedFunction`(*\*args*, *\*\*kwargs*)¶
:   A TabulatedFunction uses a set of tabulated values to define a mathematical function. It can be used by various custom forces.

    TabulatedFunction is an abstract class with concrete subclasses for more specific types of functions. There are subclasses for:

    > - 1, 2, and 3 dimensional functions. The dimensionality of a function means the number of input arguments it takes.
    > - Continuous and discrete functions. A continuous function is interpolated by fitting a natural cubic spline to the tabulated values. A discrete function is only defined for integer values of its arguments (that is, at the tabulated points), and does not try to interpolate between them. Discrete function can be evaluated more quickly than continuous ones.

    `__init__`(*\*args*, *\*\*kwargs*)¶

    Methods

    |  |  |
    | --- | --- |
    | `Copy`((self) -> TabulatedFunction) |  |
    | `__init__`(\*args, \*\*kwargs) |  |

    `Copy`(*self*) → TabulatedFunction¶

    `__delattr__`¶
    :   x.\_\_delattr\_\_(‘name’) <==> del x.name

    `__format__`()¶
    :   default object formatter

    `__getattribute__`¶
    :   x.\_\_getattribute\_\_(‘name’) <==> x.name

    `__hash__`¶

    `__reduce__`()¶
    :   helper for pickle

    `__reduce_ex__`()¶
    :   helper for pickle

    `__sizeof__`() → int¶
    :   size of object in memory, in bytes

    `__str__`¶

High performance molecular simulation on GPUs


### Navigation

- Application Layer
- Library Layer
  - Core Objects
  - Forces
  - Integrators
  - Extras

©2015, Stanford University and the Authors.
|
Powered by Sphinx 1.3.1
& Alabaster 0.7.7
|
Page source
